# The Human Health Assessment to Phthalate Acid Esters (PAEs) and Potential Probability Prediction by Chromophoric Dissolved Organic Matter EEM-FRI Fluorescence in Erlong Lake

**DOI:** 10.3390/ijerph15061109

**Published:** 2018-05-29

**Authors:** Meichen Ji, Sijia Li, Jiquan Zhang, Hui Di, Fengxu Li, Tianji Feng

**Affiliations:** 1 School of Environment, Institute of Natural Disaster Research, Northeast Normal University, Changchun 130024, China; jmc889@nenu.edu.cn (M.J.); lisj983@nenu.edu.cn (S.L.); dih717@nenu.edu.cn (H.D.); lifx144@nenu.edu.cn (F.L.); fengtj449@nenu.edu.cn (T.F.); 2 Key Laboratory for Vegetation Ecology, Ministry of Education, Changchun 130024, China; 3 State Environmental Protection Key Laboratory of Wetland Ecology and Vegetation Restoration, Northeast Normal University, Changchun 130024, China

**Keywords:** chromophoric dissolved organic matter, fluorescence, fluorescence regional integration, phthalate acid esters, health risk assessment

## Abstract

Phthalate acid esters (PAEs) are suspected to cause wide environmental pollution and have adverse effects on human health. Three priority control phthalates, namely dimethyl phthalate (DMP), diethyl phthalate (DEP), and dibutyl phthalate (DBP), were determined in 45 water samples from the largest drinking water source in Jilin Province. Chromophoric-dissolved organic matter (CDOM), which are composed of complex compounds and are a proxy for water quality, can be monitored using a fluorometer. This study attempted to understand the correlations of the CDOM fluorescence regional integration (FRI) components with PAEs and CDOM characteristics under seasonal and spatial variations in the Erlong Lake. The characteristics of the CDOM absorption parameters in different water samples showed a higher aromatic content and molecular weight in October because of increased terrestrial inputs. The Σ3PAEs concentrations ranged from 0.231 mg L^−1^ to 0.435 mg L^−1^ in water, and DEP contributed to more than 90% of the Σ3PAEs. The FRI method identified five fluorescence components: one tyrosine-like (R1), one tryptophan-like (R2), one fulvic-like (R3), one microbial protein-like (R4), and one humic-like (R5) component. However, significant relationships exist between DEP and R3 (*R*^2^ = 0.78, *p* < 0.001), R4 (*R^2^* = 0.77, *p* < 0.001), and R5 (*R*^2^ = 0.58, *p* < 0.001). Quantifying the relationship between CDOM and PAEs was highly significant, because the results will simplify the componential analysis of pollutants from a spatiotemporal perspective as compared to traditional chemical measurements. The human health risk assessment results revealed no human health risk (*HQ* < 1) in the Erlong Lake basin.

## 1. Introduction

Lakes are critical in the transportation, transformation, and storage of large amounts of carbon from terrestrial sources, and they contribute to regional effects on climate [1,2]. With the development of industry and agriculture, lakes are disturbed by anthropogenic activities, such as agricultural, industrial activities, and living organic pollutants that destroy water quality and ecological balance, and alter the carbon balance [2]. In particular, drinking water quality and the surrounding residents’ breeding industry are affected [3]. Therefore, it is necessary to study the types and distribution characteristics of pollutants and to assess the ecological and health risks for inland lakes.

Chromophoric-dissolved organic matter (CDOM), the colored fraction of dissolved organic matter (DOM), has a profound effect on underwater light penetration as CDOMs absorb slightly in the blue and ultraviolet (UV) region [3]. CDOM originates from the surrounding terrestrially imported substances (allochthonous) and from the degradation of plant by microorganisms (autochthonous) in natural water bodies. High CDOM concentrations in inland waters are often closely related to terrestrial inputs [4,5,6]. CDOMs generally show temporal and spatial distributions that are affected by the land use types, anthropogenic inputs, climate, hydrology, etc., and the environmental background could control its structure and composition [7]. Owing to the various origins, complexity, and non-conservative behavior of CDOM in water, it is difficult to quantify the dynamics and the corresponding control factors that affect the compositional variability of CDOM using traditional chemical measurements [5], such as the isotopic tracer method. Excitation-emission matrix (EEM) fluorescence spectroscopy has been widely used to provide useful information approximately the source (allochthonous and autochthonous) and the quality of CDOM [7,8,9,10]. The traditional “peak-picking” method is unreliable to evaluate CDOM dynamics in aquatic ecosystems because of the overlapped fluorophores of the CDOM EEM [7]. In recent years, EEM combined with parallel factor analysis (EEM-PARAFAC) and EEM coupled with fluorescence regional integration (EEM-FRI) techniques can provide detailed information on the environmental fluorescent-dissolved organic matter (FDOM) dynamics in aquatic ecosystems. The EEM-PARAFAC technique can provide only several paired fluorescence intensity data with limited FDOM component information [11]. When compared to the EEM-PARAFAC method, EEM-FRI as a quantitative technique is associated with all of the wavelength-dependent paired fluorescence intensity data in an EEM. EEM-FRI has been proven to be an effective method to integrate the volumes beneath the defined EEM regions, while the integrated fluorescence intensity represents an integral part of the FDOM. Chen et al. 2003 [12] introduced FRI to the EEM spectra and used consistent excitation and emission wavelength boundaries to separate an EEM into five regions, which were related to different FDOM components: R1, tyrosine-like component; R2, tryptophan-like component; R3, fulvic-like component; R4, microbial protein-like component; and, R5, humic-like component. Chen et al. 2003 [12] used EEM-FRI to investigate the composition and the transformation of humic and fulvic acids from landfill. Zhao et al. (2017) [11] used fluorescence EEM and regional integration techniques to characterize CDOM in Chinese river waters. However, the relationships between specific pollutants and CDOM have not been investigated in the past. Based on the rapid and simple advantages of using a fluorometer for an on-site determination of CDOM fluorescence in the water, the discussion of the relationship between specific pollutants and CDOM is important, which signifies an application prospect of dynamic monitoring and health assessment. In this study, the relationship between the components of CDOM by EEM-FRI and PAEs was conducted. Further using remote sensing technology to assess the health risk for PAEs should satisfy the need for a large-scale and low-cost method [6].

Phthalates esters (PAEs) are synthetic compounds that are used primarily as plasticizers, which can be used to improve the durability, elasticity, and plasticity of polyvinyl chloride (PVC) products [13]. PAEs are common additives in paints, lubricants, adhesives, insecticides, packaging, and cosmetics, and are therefore continually released into the environment and are ubiquitous in water. Some PAEs have reproductive and developmental toxicity in animals and bio-accumulates in fats, and have been shown to be carcinogenic and estrogenic [13]. Furthermore, the hydroxylation of PAEs and the alkyl chain length significantly affect their ability to bind to peroxisome proliferator-activated receptors and estrogen receptors [14]. Previous studies have found that PAEs are ubiquitous in many types of environmental samples, e.g., air, water, soils, vegetables, and sediments [14,15,16]. Wang et al. (2017) [14] reported that the human health risks of PAEs were non-cancer risks and showed that the negligible but carcinogenic risk of DEHP (diethylhexyl phthalate) exceeded the threshold limit value in agricultural soils. The risks that are posed by PAEs from drinking water on human health still exist [16].

The Erlong Lake is the drinking source of Siping city, Northeast plain of China. It has an irrigation area of approximately 6700-hm^2^ cultivated land, which is the largest reservoir in the Jilin province. The increase agricultural activities, industrial wastewater, and domestic sewage discharge contributed to the increased pollution of Erlong Lake. The water quality of the Erlong Lake is closely related to the health of residents, growth of crops, fisheries, and ecosystems in the region. To maintain agricultural yields and activities, agricultural plastic films, fertilizers, and pesticides are used, which introduces more PAE contaminants to the lake. Subsequently, various farming activities, the location of the sewage discharge outlets, and residential density cause spatial variations in the PAEs in water, which may lead to differences in exposure risk levels. Nevertheless, the issue of pollution from PAE compounds has not been given enough attention. Ensuring safe drinking water for human health is a global problem [17].

Accordingly, the objectives of this study are to investigate: (i) the temporal and spatial distributions and variations in selected PAE compounds in the Erlong Lake, and assess the risks to human health, (ii) the sources and compositions of FDOM by the EEM-FRI method, and (iii) the relationship between five EEM-FRI-divided FDOM components and PAEs in surface waters, and discuss the potential probability in using CDOM fluorescence as a tool for predicting the concentrations of PAEs. These results provide a basis for the evaluation of health risk based on remote sensing. Similarly, quantitative assessments and the monitoring of PAEs in the Erlong Lake provide an early pollution warning and management measures for water sources conservation.

## 2. Materials and Methods

### 2.1. Study Area and Water Sampling

The Erlong Lake (43°7′45″–43°20′34″ N, 124°46′1″–124°58′29″ E) is located in the east Siping City of the Jilin Province, China. The east Liaohe River is the primary supply source and pollution source of the Erlong Lake. The lake area above the dam site is 3799 km^2^ and it is located in the middle and upper reaches of the east Liaohe River basin. The water surface of the Erlong Lake spans two provinces, three cities and five counties, and is the water source of the residents of Siping city and the irrigation source for approximately 6700 hm^2^ of cultivated land. The total storage capacity is 1.762 billion m^3^, and the average flow rate of the designed flow is 10,100 m^3^/s. The Erlong Lake is located in the transitional zone of humid climate and semi-humid climate with an average air temperature of 5.8 °C and annual precipitation of 650 mm [18].

**Figure 1:**
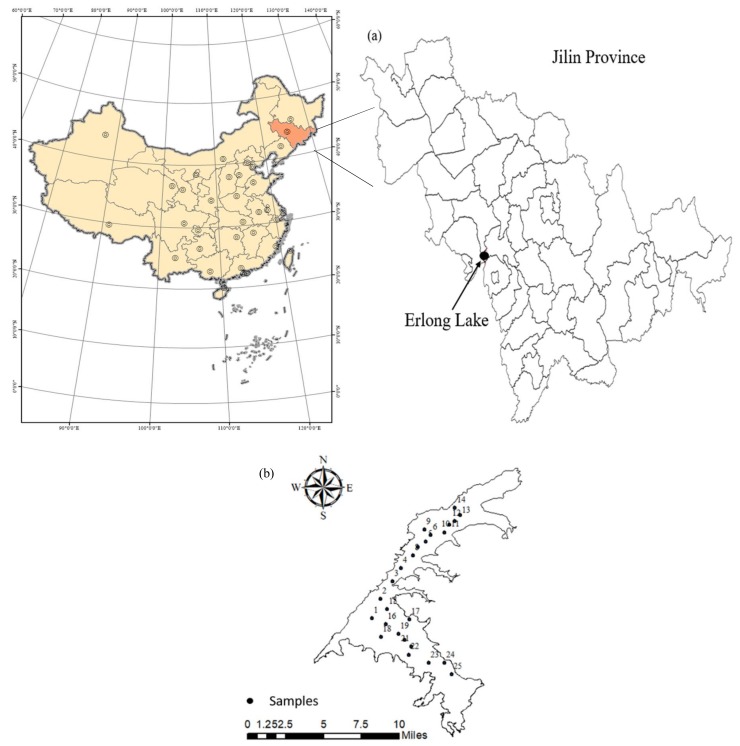
(**a**) Location of the Erlong Lake, Jilin Province, Northeast China. (**b**) The sampling sites among the 25 samples of the Erlong Lake.

A total of 45 water samples that were collected at 25 points on the lake surface were recorded by a global positioning system (Figure 1). The detailed sampling location information regarding each field survey from the various sampling points is shown in Figure 1. The sampling courses were performed in June (normal season) and October (dry season) in 2017. The water samples were collected using acid-cleaned Niskin bottles, and subsequently the water sample was placed in a brown bottle. At each point, 4 L water samples were collected from a depth of 0–0.2 m from the surface. The collected samples were stored at 4 °C in coolers and were subsequently transported to the laboratory within 3–5 h. The physical and chemical parameters were determined within 24 h.

### 2.2. Water Quality Determination

The water quality parameters, electrical conductivity (EC) and pH, of each water sample were measured onsite using a portable multiparameter water quality analyzer (Hach, Loveland, CO, USA). By referring to the Environmental Quality Standards for Surface Water (GB3838-2002, China) (http://kjs.mep.gov.cn/), chemical oxygen demand (COD) was determined using dichromate, ammonia nitrogen (NH_3_-N) via Nessler’s reagent colorimetry, and total phosphorous (TP) using the molybdenum blue method after the samples were digested with potassium peroxydisulfate [19]. To determine the dissolved organic carbon (DOC) concentrations, all of the water samples were filtered through a pre-combusted Whatman GF/F (1825-047) filter (0.45 μm) under low vacuum and were measured by the Shimadzu TOC-VCPN analyzer. Chlorophyll a (Chl-a) was extracted from the filtered (0.45-µm Whatman GF/F) sample using a 90% acetone solution; subsequently, the concentrations were determined using a UV spectrophotometer (UV-2006 PC, Shimadzu, Kyoto, Japan); the detailed process can be found in Song et al. (2012) [20]. The total suspended matter (TSM), inorganic suspended matter (ISM), and organic suspended matter (OSM) were determined by gravimetrical analysis. Detailed descriptions about this analysis are available in Song et al. (2012) [19].

### 2.3. Phthalate Acid Esters 

#### 2.3.1. Sample Extraction and Chemical Analysis

The water samples were extracted using a classical soil-phase extraction method [15]. Water samples of 2 L had previously been acidified to pH 7 by hydrochloric acid, using OASIS HLB to enrich the PAEs in water. Subsequently, 5% methyl alcohol was used to clean the column, 9-mL of a mixed solution of 95% diethyl ether, and 5% methyl alcohol was used to elute the sample with the flow velocity of 1 mL min^−1^. Finally, the remains were reduced to 1 mL under gentle nitrogen flow. Prior to the instrumental analysis, the 0.05 ng internal standard was added to the sample.

Quantification of the PAEs was performed on the GCMS-QP 2010 Plus gas chromatography–mass spectrometry system (Shimadzu Corporation, Kyoto, Japan). A DB-5 MS (30 m × 0.25 mm i.d.; 0.25-μm film thickness, Agilent, Santa Clara, CA, USA) fused-silica capillary column was used to separate the target compounds. The column temperature program was initiated at 60 °C for 1 min and increased to 220 °C at a rate of 20 °C/min, held for 1 min, and finally to 280 °C for 5 min and held for 8 min. The carrier gas helium (99.999% purity) was flowed at a constant flow rate of 1.0 mL min^−1^. A 1-μL sample was injected in the splitless mode. The temperatures of the injector, each extract, the GC-MS transfer line, and the post run were 250 °C, 250 °C, 280 °C, and 285 °C, respectively. These were operated in the electron impact mode (70 eV).

#### 2.3.2. Quality Analysis and Quality Control

An appropriate concentration of the standard solution was spiked into each sample to estimate the recovery and the performance of the methods. During the analysis, a procedural blank, a matrix-spiked sample, and duplicates were processed for each batch of the water samples. The recoveries from the standard mixture solution of three PAEs, including dimethyl phthalate (DMP), diethyl phthalate (DEP), and dibutyl phthalate (DBP) (50 ng L^−1^ each) ranged from 76.3% to 108.6% in the spiked water. Each water sample was analyzed in triplicate with relative standard deviations of less than 23%. The method determination limit of the PAEs ranged from 0.08 ng L^−1^ to 0.51 ng L^−1^.

### 2.4. Chromophoric-Dissolved Organic Matter Absorbance and Three-Dimensional Fluorescence

The water samples were first filtered under low vacuum through a pre-combusted Whatman GF/F (1825-047) filter (0.7-µm pore size), and subsequently, through a pre-rinsed 25-mm diameter Millipore membrane cellulose filter (0.22-µm pore size) into brown glass bottles [6]. The Shimadzu UV-2600 spectrophotometer with 1-cm quartz cells between 200 nm and 800 nm at 1 nm increments with Milli-Q water as a reference was used to measure the twice-filtered water samples [20]. The CDOM absorption coefficient *α*_CDOM_($\lambda^{\prime }$) was calculated from the measured optical density (OD), as follows:

(1) $$\alpha_{\mathrm{CDOM}}\left(\lambda^{\prime }\right)\;=\;2.303\mathrm{OD}\left(\lambda \right)/\gamma$$

where *γ* is the cuvette path length (0.01 m) and 2.303 is the conversion factor. OD(*λ*) is the optical density at the same wavelength [6].

The CDOM EEMs were obtained using the Hitachi F-7000 fluorescence spectrometer (Hitachi High-Technologies, Tokyo, Japan) with a 700-V xenon lamp as the light source [20]. The wavelength ranges of excitation were 220–450 nm at 5-nm sampling interval and 250–600 nm for the emission at a 1-nm sampling interval, with the scanning speed being maintained at 1200 nm/min. The spectrum of the Milli-Q water recorded as the blank was subtracted from all of the EEMs to eliminate the water Raman scatter peaks [21]. To eliminate the inner-filter effect and the Raleigh scattering, the EEM fluorescence spectra need to be corrected for absorbance; more details can be found in Li et al., 2017 [6].

The fluorescence indices *FI*_370_ and *FI*_310_ were used to characterize the CDOM source. *FI*_370_ was defined by McKnight et al. (2001) [22] as the ratio of the excitation fluorescence intensity to that of the emission intensity, as follows: *E_x_/E_m_* = (370/450 nm)/(370/500 nm) [2], to distinguish between terrestrially (*FI*_370_ < 1.4) and microbially (*FI*_370_ > 1.9) derived fulvic acids. To determine the contribution of the autochthonous, *FI_310_* is defined as the ratio of excitation and emission intensities, as follows: *E_x_*/*E_m_* = (310/380 nm)/(310/430 nm) [2]. When the fluorescence index *FI*_310_ is lower than 0.7, it indicates the low levels of autochthonous CDOM components in water bodies. When the fluorescence index *FI*_310_ is higher than 0.8, it indicates the large amounts of autochthonous CDOM components in the water bodies due to the biological activity. When the fluorescence index *FI*_310_ is between 0.7 and 0.8, it implies intermediate autochthonous CDOM components [2,23].

### 2.5. Fluorescence Regional Integration (FRI)

Fluorescence regional integration (FRI) using consistent excitation and emission wavelength boundaries to divide the EEM spectra into five regions: R1, tyrosine-like component (*E_x_*: 200–250 nm; *E_m_*: 280–330 nm); R2, tryptophan-like component (*E_x_*: 200–250 nm; *E_m_*: 330–380 nm); R3, fulvic-like component (*E_x_*: 200–250 nm; *E_m_*: 380–550 nm); R4, microbial protein-like component (250 < *E_x_* < 400 nm; *E_m_*: 280–380 nm); and, R5, humic-like component (250 < *E_x_* < 400 nm; *E_m_*: 380–550 nm) [11]. The integral volume (*Φ_i_* or *F_Ri_*) can be expressed, as follows:

(2) $$F_{Ri}\left(\Phi_{i}\right)\;=\;\underset{ex}{\sum }\underset{em}{\sum }I\left(\lambda_{ex}\lambda_{em}\right)\Delta \lambda_{ex}\lambda_{em},$$

where cumulative volume beneath the EEM (*Φ_i_)* at five regions (*i* = 1, 2, 3, 4, and 5) of FRI-extracted FDOM components could be presented by *F_Ri_ (Φ_i_)* (*i* = 1, 2, 3, 4, and 5), further was simplify exhibited by *F_Ri_* (*i* = 1, 2, 3, 4, and 5). For each lake sample, the *F_Ri_* (*i* = 1, 2, 3, 4, and 5) at five regions (*i* = 1, 2, 3, 4, and 5) was calculated. Then, the cumulative volume beneath the EEM (*Φ_T_*) was calculated as *Φ_T_* = ∑*Φ_i_* (*i* = 1, 2, 3, 4, and 5), which was presented by *F*_SUM_ (unit: nm). Δ*λ_ex_* is the internal excitation wavelength (taken as 5 nm), Δ*λ_em_* is the internal emission wavelength (taken as 1 nm), and *I* (*λ_ex_ λ_em_*) represents the fluorescence intensity at the corresponding wavelength of five regions that are divided by EEM-FRI. More details could be found in Chen et al. (2003) [12]. The allochthonous substances *F_R(3+5)_* consisted of fulvic-like components F_R3_ and humic-like components *F_R5_*. The autochthonous substances *F_R(1+2+4)_* consisted of tyrosine-like component *F_R1_*, tryptophan-like component *F_R2_*, and microbial protein-like component *F_R4_*. [11].

### 2.6. Health Risk Assessment

By referring to the widely applied risk assessment guidelines that are recommended by the United States Environmental Protection Agency (USEPA) (2013) [14,15], this study assesses the potential health risk to inhabitants, as well as the non-cancer and carcinogenic risks of PAEs. The non-carcinogenic risk is considered by the hazard quotient (*HQ*) determined by the average daily dosage (*ADD*, mg kg^−1^ day^−1^) and the reference dosage (*RfD*, mg kg^−1^ day^−1^) to represent the exposure pathway of the intake, ingestion, dermal absorption, and inhalation. The formulas are the following:

(3) $$HQ\;=\;\frac{ADD}{RfD}$$

(4) $$ADD\;=\;\frac{\left(C\times DR\times EF\times ED\right)}{\left(BW\times AT\right)}$$

(5) $$HI\;=\;\sum^{\text{​}}HQ\;=\;\sum^{\text{​}}\frac{ADD}{RfD},$$

where *C* is the contaminant concentration (mg kg^−1^ d^−1^), *DR* is the daily consumption rate (L d^−1^), *EF* is the exposure frequency (d year^−1^), *ED* is the exposure duration (year), *BW* is the body weight (kg), and *AT* is the average lifetime exposure (d). *RfD* is defined as the daily maximum permissible level of pollutants. *RfD* and *ADD* were collected from the integrated risk information system (IRIS) database that was developed by the EPA. The hazard index (*HI*) was calculated to assess the non-cancer risk by the level comparison of the PAE compounds. It is considered that *HI* > 1 indicates a high non-cancer risk, while *HI* < 1 indicates a low non-cancer risk.

The carcinogenic risk is considered by the carcinogenic risk (*CR*) (unitless), as follows:

(6) CR = ADD×CSF,

(7) $$RI\;=\;\sum^{\text{​}}CR\;=\;\sum^{\text{​}}\left(ADD\times CSF\right),$$

where CFS is the slope factor of the carcinogen ((mg kg^−1^ d^−1^)^−1^). The risk index (*RI*) < $1\times 10^{-6}$ indicates a very low carcinogenic risk, whereas $RI>1\times 10^{-4}$ means an unacceptable risk level.

## 3. Result and Discussion

### 3.1. Water Chemistry

The spatial-temporal variations in water quality are related to land use and land cover [24]. When all of the water samples were pooled together in the Erlong Lake, a significant seasonal variability of water quality parameters occurred, i.e., pH, EC, DOC, Chl-a, TP, NH_3_-N, COD, ISM, and OSM for the 45 water samples are displayed in Table 1. The pH in June (average ± SD, 8.0 ± 0.26) is higher than that in October (average ± SD, 7.8 ± 0.04), which was associated with a high EC (average ± SD, 463.616.76 µS cm^−1^) in June and a lower EC (average ± SD, 349.814.75 µS cm^−1^) in October. DOC represents an essential link between terrestrial and aquatic ecosystems [25,26]. The average DOC concentration in October (23.63 mg/L) is lower than that in June (26.38 mg/L). Likewise, a higher Chl-a, COD, ISM, and OSM were also found in water samples in June than in October. The average Chl-a concentration in June (39.01 μg L^−1^) is higher than that in October (14.51 μg L^−1^); further, during the sampling period, many floating algae appeared on the lake surface. This result indicated the higher contribution of algal and microbial activity in June, which is related to the increased temperature and sunlight in the summer [25]. However, low nutrients, i.e., TP and NH_3_-N concentration, were exhibited in June (Table 1). This may be associated with the higher precipitation in the summer owing to the dilution effect. COD is related to the organic pollution levels; the average COD concentration in June (28.28 mg L^−1^) is higher than that in October (22.57 mg L^−1^), signifying more human and farming activities in June. The differences were assessed statistically (ANOVA), and the *p* value for each water quality parameter in June and October was lower than 0.05.

**Figure 2:**
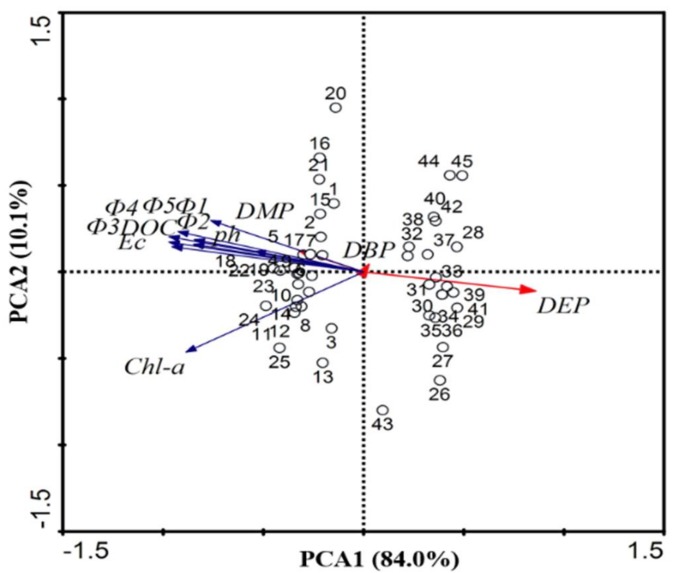
Principal component analyses (PCA) of the average values of the water quality parameters (electrical conductivity (EC), Chl-a, DOC, dimethyl phthalate (DMP), diethyl phthalate (DEP), and dibutyl phthalate (DBP)) in Erlong Lake.

PCA analysis (CANOCO 4.5 for Windows) of the average values of seven water quality parameters and FRI fluorescence component were performed for the Erlong lakes (Figure 2). The first two PCA axes (PC1, 84.0%; PC2, 10.1%) explained 94.1% of the total variability in the data set of all the average values of water quality parameters and FRI fluorescence component among the 45 samples. The first PC1 axis showed relatively high positive loadings for DEP and DBP. The DOC, EC, pH, DMP, and FRI fluorescence component had relatively high positive loadings on the second PCA axis. Most of the water samples collected (sample points from 1 to 25) in Jun were clustered on the negative side of PC1, whereas the water samples that were collected (sample points from 26 to 45) in October were scattered on the positive side of PC1. The results of PCA further illustrated that the average values of the water quality parameters were different between the June and October. The varying water quality would have an impact on the distribution of the fluorescence intensities of CDOM components.

The units of dissolved organic carbon (DOC), total phosphorous (TP), ammonia nitrogen (NH_3_-N), chemical oxygen demand (COD), inorganic suspended matter (ISM), and organic suspended matter (OSM) are mg L^−1^, the units of Chlorophyll a (Chl-a) is µg L^−1^. *N* represents the number of samplings. Water quality standard i grade, COD ≤ 15, NH_3_-N ≤ 0.15, and TP ≤ 0.02; Water quality standard ii grade, COD ≤ 15, NH_3_-N ≤ 0.5, and TP ≤ 0.1; Water quality standard iii grade, COD ≤ 20, NH_3_-N ≤ 1.0 and TP ≤ 0.2; Water quality standard iv grade, COD ≤ 30, NH_3_-N ≤ 1.5, and TP ≤ 0.3; Water quality standard v grade, COD ≤ 40, NH_3_-N ≤ 2.0 and TP ≤ 0.4.

### 3.2. Temporal and Spatial Variations of Phthalate Acid Esters 

**Figure 3:**
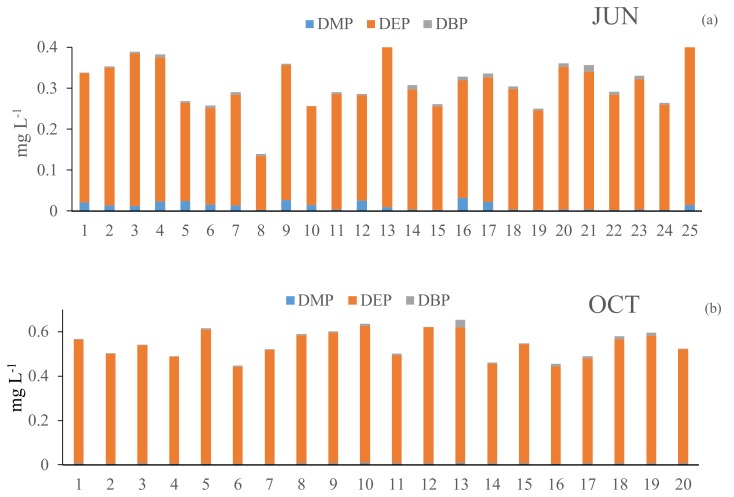
Spatial distributions of three PAEs from the Erlong Lake in June (**a**) and October (**b**) 2017.

The distributions of the three US EPA priority PAEs (Σ3PAE, including dimethyl phthalate (DMP), diethyl phthalate (DEP), dibutyl phthalate (DBP)) in the Erlong Lake from the sampling sites were investigated in June and October. The results of the relative contributions of the three PAE congeners are presented in Table 2 and Figure 3. The mean concentrations of DBP detected in the present study were well above the reference dose (*RfD*: 3 µg L^−1^), and is regarded as unsafe in China for surface water (Environmental Quality Standard for Surface Water of China, GB3838-2002) (Table 2). The Σ3PAEs concentrations ranged from 0.231 mg L^−1^ to 0.435 mg L^−1^, and the arithmetic mean is 0.318 mg L^−1^, from 0.447 mg L^−1^ to 0.654 mg L^−1^, and the arithmetic mean is 0.546 mg L^−1^ for June and October, respectively. Among the three PAEs that were detected in the Erlong Lake in June, DMP, DEP, and DBP were detected in all of the samples at average concentrations of 0.013, 0.299, and 0.006 mg L^−1^, respectively; in October, the average concentrations of DMP, DEP, and DBP were 0.006, 0.533, 0.007 mg L^−1^, respectively. The value of DEP was significantly higher in October than in June (ANOVA, *p* < 0.05); this phenomenon was caused by the seasonal hydrology and temperature [16]. The seasonal average of DMP, DEP, and DBP concentrations were in the order of 0.01, 0.403, and 0.007 mg L^−1^. When considering the individual PAE congeners in this area, the results show that DEP is the most abundant in the water samples, which is the major component of three PAEs, contributing to 95.9% (Table 2 and Figure 3). The next most dominant PAE was DMP, contributing to 2.4%, followed by DBP, contributing 1.7% of the ∑3PAEs concentrations in all 45 water samples. The differences were assessed statistically (ANOVA), and the *p* value for DEP and DBP in June and October were lower than 0.05.

As shown in Figure 3, significant variations in DMP, DEP, and DBP occurred at different sampling points in June and October. It is noteworthy that the PAE levels may be related to the types of local waste discharge, such as sewage water, food packaging, and scrap material during the sampling period. In addition, obvious differences were found in the proportions of DMP and DBP to the total PAEs during the two periods, indicating various possible sources of PAEs. A previous study has shown the positive correlation between the concentrations of PAEs with the low-molecular-weight DEP and DMP; further, the agricultural runoff and the solubility of DMP and DEP indicate their potential abundance in water (dissolution phase) [27]. DBPs are used in epoxy resins and special adhesive formulations, indicating that the PAEs at the sampling locations are from industrial pollution [16]. The concentration of DBP and DEP were 0.012 mg L^−1^ and 0.033 mg L^−1^ at the 13th point in October and June, respectively. The 13th sampling point that was near the sewage discharge outlet and the values of DBP and DEP at point of 13 were higher than in other sampling sites, indicating that the source of DBP and DEP were from the allochthonous. In general, the results show that the spatial distributions of PAEs in the Erlong Lake were site specific.

### 3.3. Fluorescence Regional Integration-Divided Fluorescent-Dissolved Organic Matter Components

**Figure 4:**
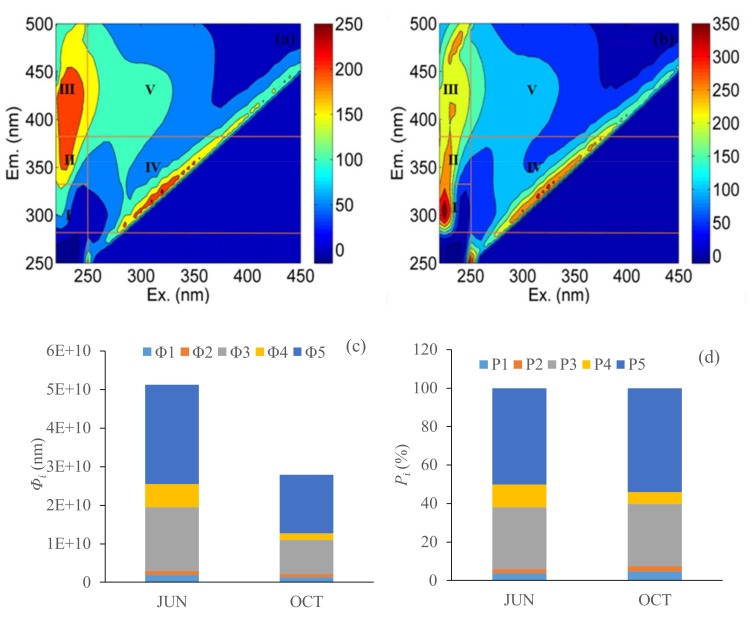
Excitation-emission matrix (EEMs) of water samples, point 1 (**a**) and point 17 (**b**) from the Erlong lake in October, (**c**) distributions of fluorescence regional integration (FRI)-extracted fluorescent-dissolved organic matter (FDOM) components, and (**d**) distributions of percentages of FRI-extracted FDOM components.

FRI has been widely used to quantitatively analyze and study the EEM fluorescence characteristics of CDOM [11,28]. The total fluorescence intensities, the fluorescence intensities of five components, and the relative contributions of different components to the total fluorescence intensities *P_i_* showed seasonal variations. As shown in Figure 4a,b, EEM was divided into five regions by FRI, each representing a CDOM fluorescence fraction: the tyrosine-like (R1), tryptophan-like (R2), microbial protein-like (R4), fulvic-like (R3), and humic-like (R5) fluorescent components [11,29]. The excitation-emission area volumes *Φ_i_* and *P_i_* (*i* = 1, 2, 3, 4, 5) were the proportion of the total fluorescence intensities and the relative contributions of five different components to the total fluorescence intensities, respectively. As shown in Figure 3a,b, the total fluorescence intensities *F_SUM_* decreased from 5.1 × 10^10^ to 2.8 × 10^10^ nm in June and October. The fluorescence intensities *Φ*_5_ account for 50% in June and 54% in October of the total fluorescence intensities. The fluorescence intensities *Φ*_3_ and *Φ*_5_ were dominant in the two months and they were from the allochthonous. The value of *P*_(3+5)_ was increased from 82.4% in June to 86.4% in October, indicating more allochthonous substances in October.

### 3.4. Chromophoric-Dissolved Organic Matter Absorption and Fluorescence Characteristic

The composition and distributions of CDOMs were impacted by the environmental factors (water quality, soil, vegetation, land use, and rainfall) and could generally affect the absorption and fluorescence of CDOMs at certain wavelengths [20]. Generally, the absorption coefficient *a*_CDOM_(350) is used to characterize the concentration of CDOMs [7,29] and *a*_CDOM_(254) can be used as a proxy for characterizing the aromaticity of CDOMs [7,30]. The average *a*_CDOM_(350) and *a*_CDOM_(254) in October have exhibited significantly higher values than in June (Table 3). The spectral slopes *S*_275–295_ and *E*_250:365_ [*a*_CDOM_(250)/*a*_CDOM_(365)] could be used as an indicator for terrigenous DOC percentage, which were used to track the changes in CDOM molecule size [31]. The *S*_275–295_ values (0.24 ± 0.12 nm^−1^) in October were lower than those in June (0.26 ± 0.10 nm^−1^), and they exhibited a consistent tendency of *E*_250:365_ and *S*_275–295_ (ANOVA, *p* < 0.05). This indicated that the increase in aromatic compounds and the percentage of high-molecular-weight fulvic acid of CDOMs in October was greater in October. The high *E*_250:365_ values are associated with the low content of aromatic hydrocarbon and the molecule weights were remarkable in June. These results that are associated with the higher contribution from algal and microbial activity in June are consistent with the results of water quality presented in Table 1.

**Figure 5:**
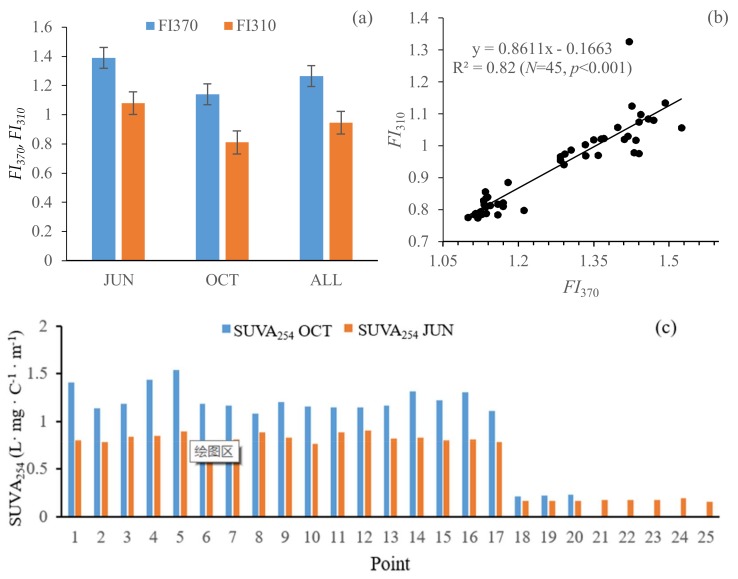
(**a**) Seasonal fluorescence indices (*FI*_370_ and *FI*_310_), (**b**) correlation between fluorescence indices *FI*_370_ and *FI*_310_, and (**c**) spatial and seasonal variations of the *SUVA*_254_ from all 45 water samples in the Erlong Lake.

As an effective index to characterize the DOC concentration and DOM aromaticity, we used *SUVA*_254_ values with the ratio of *a*_CDOM_(254)/DOC [30,31,32]. Higher *SUVA*_254_ values indicate aquatic systems with abundant vascular plant inputs, and the allochthonous sources dominated the organic matter content [31,32,33]. Meanwhile, the lower values indicate more autochthonous sources (algal and microbial). As shown in Table 3, the average value of *SUVA*_254_ was higher in October than in June (ANOVA, *p* < 0.05). Relatively lower *SUVA*_254_ measurements were found from point 18 to point 25, indicating that the aromatic moieties of CDOM in this environment were lower when compared with the other points in June and October (Figure 5c). In June and October, the *SUVA*_254_ value was higher in point 5, and the values of *a*_CDOM_(254) were the highest at 22.57 m^−1^ and 28.71 m^−1^ with the mean values of 16.06 m^−1^ and 23.04 m^−1^ (ANOVA, *p* < 0.05), respectively. Meanwhile, the lower value of DOC concentrations were 25.30 mg L^−1^ and 18.65 mg L^−1^, with the mean values of 26.08 mg L^−1^ and 21.73 mg L^−1^, respectively. According to the Li et al. (2016) [25], point 5 had a lower color DOC concentration that was resulting from the algal-derived DOC in the waters.

As shown in Table 3 and Figure 5a, the average values of the fluorescence index *FI*_370_ is 1.39 in June and 1.14 in October (ANOVA, *p* < 0.05); the average values of the fluorescence index *FI*_310_ is 1.04 in June and 0.81 in October (ANOVA, *p* < 0.05). In June, the average value of *FI*_310_ was above 0.8 and *FI*_370_ was approximately equal to 1.4, indicating that CDOM sources were derived from the autochthonous; in October, the average value of *FI*_310_ is higher than 0.8 and *FI*_370_ is less than 1.4; the primary origin of CDOM is the terrestrial humic-like substances. A positive linear relationship was also found between *FI*_370_ and *FI*_310_ (*R*^2^ = 0.82, *p* < 0.01, *N* = 45) (Figure 5b), showing that *FI*_310_ and *FI*_370_ represented a similar indication in CDOM sources. This result is consistent with the findings of Zhao et al. (2017) [2].

### 3.5. Fluorescence Regional Integration Fluorescence Component Versus Chromophoric-Dissolved Organic Matter

As shown in Table 4, significant positive correlations exist between *F*_R_ for fulvic-like components R3, microbial protein-like R4, and humic-like components R5 (*p* < 0.01), indicating that they may originate from similar sources. However, no strong correlations were found for the tyrosine-like R1, the tryptophan-like R2, and the microbial protein-like R4 for the water samples in the Erlong Lake. The tryptophan-like components *F*_R2_ showed a medium positive correlation with the tyrosine-like *F*_R1_ (*R*^2^ = 0.68, *N* = 45), fulvic-like components *F*_R3_ (*R*^2^ = 0.67, *N* = 45), and humic-like components *F*_R5_ (*R*^2^ = 0.69, *N* = 45), indicating that parts of these *F*_R1_ fluorescent components were likely from some common sources. Significant positive correlations were found between *F*_R3_, *F*_R4_, and *F*_R5_ (*R*^2^ > 0.88, *p* < 0.01).

### 3.6. Fluorescence Regional Integration Fluorescent Components Versus Water Quality Parameters

As shown in Table 5, significant correlations were exhibited between DOC and *F*_R_ for the fulvic-like R3 (*R*^2^ = 0.68, *p* < 0.01; *N* = 45), the microbial protein-like R4 (*R*^2^ = 0.68, *p* < 0.01; *N* = 45), and the humic-like R5 (*R*^2^ = 0.58, *p* < 0.01; *N* = 45), respectively. Likewise, Chl-a also showed medium relationships with *F*_R_ for the fulvic-like R3 (*R*^2^ = 0.57, *p* < 0.01; *N* = 45), and the microbial protein-like component R_4_ (*R*^2^ = 0.50, *p* < 0.01; *N* = 45). It demonstrated that some DOCs, Chl-a, and CDOMs from the water samples in the Erlong Lake stemmed from the fulvic-like and microbial protein-like component sources. The presence of microbial protein-like component R4 in the Erlong Lake samples was similar to the degradation of phytoplankton releases and humus components that were caused by microbial activities [9] and microbial oxidized components [34]. The fulvic-like R3 were derived from terrestrial substances from the soil, wetlands, or agricultural sites.

Notably, medium correlations exist between *a*(350) and *F*_R_ for the fulvic-like R3 (*R*^2^ = 0.57, *p* < 0.01; *N* = 45), the microbial protein-like component R4 (*R*^2^ = 0.52, *p* < 0.01; *N* = 45), and humic-like R5 (*R*^2^ = 0.55, *N* = 45) (Table 5). These results imply that the CDOM with humic-like and microbial protein-like components were from a common source in the lake. However, a relatively weak relationship was revealed between *a*(350) and *F*_R_ for R1 and R2, respectively. In summary, based on such discrepancies, we speculate that the terrigenous pollutants and autochthonous tryptophan-like substances that were caused by microbial activities were important factors regulating the migration and transformation of CDOMs in the Erlong Lake.

### 3.7. Fluorescence Regional Integration Fluorescent Components Versus Phthalate Acid Esters 

**Figure 6:**
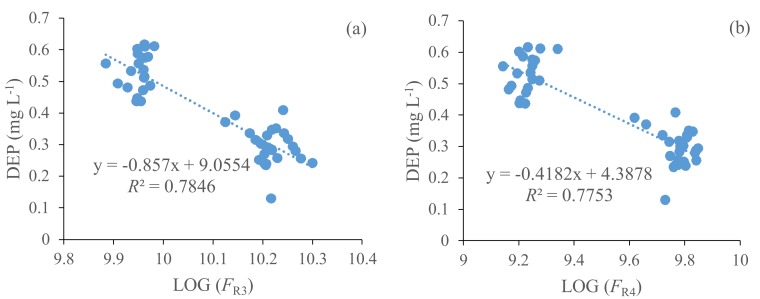
Correlation between (**a**) FRI fluorescence component 3 and diethyl phthalate (DEP), (**b**) FRI fluorescence component 4 and DEP.

When all the samples were pooled together (*N* = 45), a correlation also existed between the FRI fluorescent components and PAEs congeners, as shown in Table 6. As shown in Figure 6, in the Erlong Lake, R3 and DEP showed a significant correlation with the correlation coefficient of 0.78 (two-tailed, *p* < 0.001). Likewise, R4 (*R^2^* = 0.77, *p* < 0.001), and R5 (*R^2^* = 0.58, p < 0.001) also showed a significant correlation with DEP, respectively. ∑3PAEs and R3, R4, and R5 showed significant correlations with correlation coefficients of 0.76 (two-tailed, *p* < 0.001), 0.75 (two-tailed, *p* < 0.001), 0.58 (two-tailed, *p* < 0.001), respectively. We speculated that the CDOMs and PAEs underwent chemical adsorption and contained fluorophore; consequently, they had similar origins to the waters that were affected strongly by terrestrial sewage inputs and agricultural insecticides. The result shows a positive correlation between the concentrations of PAEs with DEP and the agricultural soil runoff. It shows the contribution of terrestrial fulvic-like fluorophores, humic-like fluorophores, and microbial protein-like to R3, R4, and R5. The solubility of DEP indicates the reason for their potential enrichment in water and with extremely low production and consumption capacity [26]. PAEs contributed part of the CDOM absorption in natural surface waters because a small amount of organic matter was present. Although the results were limited by the conditions of the single research area, they may provide an overall situation of PAEs that are associated with FRI fluorescent components. Based on the correlation between DEP, R3, and R4 of water samples that were collected in the Erlong Lake, the CDOM fluorescence using a fluorometer has the potential probability to monitor PAEs in natural surfaces. However, it requires large amounts of samples for verifications and adjustments in different regions. In the future, we will continue studying the chemical transformation between humic-like, fulvic-like, and PAEs.

### 3.8. Health Risk Assessment

According to the results above, the concentration of DBP in Erlong Lake were higher than the recommended drinking water limit (i.e., the level present in public water supplies must not exceed the drinking water standard: 3 µg L^−1^ for China, GB3838-2002). The values of the parameters for non-cancer risks can be referred from the US EPA (2013), Kong et al. (2017) [16], and Wang et al. (2015) [35]. Assuming a daily water consumption rate of 2 L and an average body weight of 60 kg for adults, the values of EF, ED, and AT were 350 d year^−1^, 30 years, and 26,280 days, respectively. The RfD values of DBP and DEP via drinking water from the Erlong Lake was estimated to be 100 µg kg^−1^ day^−1^ and 800 µg kg^−1^ day^−1^, respectively by the US EPA. The RfD value of DMP was 1000 µg kg^−1^ day^−1^ according to Wang et al. 2015 [35]. Among the individual PAE congeners that were studied, DBP, DEP, and DMP were recognized as non-cancer compounds. The non-carcinogenic risk assessment is based on a nonlinear model. In this study, the non-carcinogenic risk of PAE compounds to local inhabitants through the dietary route was evaluated. The *HQ* was employed to assess the non-carcinogenic risk in this study.

**Figure 7:**
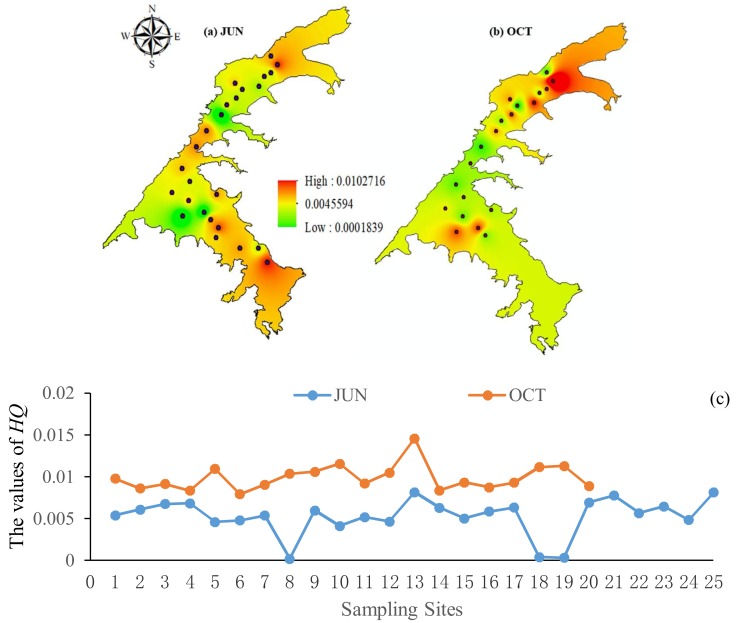
Total hazard quotient (HQ) of three PAEs in June (**a**) and October (**b**) 2017 were determined in ArcGIS 10.0, and (**c**) spatial and seasonal distributions of PAEs’ hazard quotient of sampling sites.

As shown in Figure 7, higher values were observed in October and a higher *HQ* value was observed at site 13. All of the non-cancer risks from the PAE values that were estimated in this study were far below the recommended limits (*HQ* < 1) in the two months. Therefore, no human health risks by PAE compounds occurred in the Erlong Lake. However, PAEs are partially metabolized by organisms, and future experiments will be focused on the factors affecting the concentration of PAEs in water and the relationship between the fluorescence component of CDOMs and PAE congeners.

## 4. Conclusions

The Erlong Lake is the largest drinking source of the residents of Siping City in the Jilin Province, and the irrigation area of cultivated land is approximately 6700 hm^2^. The complex optical properties of the Erlong Lake were caused by the large amounts of pollutants and dissolved constituents. This study exemplified the spatial and the temporal characteristics of three PAEs congeners and the CDOM characteristics, based on the *FRI* fluorescent components of 45 water samples that were collected in 2017. The Σ3PAEs concentrations ranged from 0.231 mg L^−1^ to 0.435 mg L^−1^, and DEP accounted more than 90% of ∑3PAEs in this lake. In October, lower CDOM optical parameters of *a*_CDOM_(254), *E*250:365, *S*_275–295_, and *S*_R_ signified the increased aromatic content and the molecular weight of CDOM. A positive correlation existed between the CDOM FRI fluorescent components and DEP (two-tailed, *p* < 0.001), and they were affected by the individual samples with high PAEs. Based on the HQ, PAE compounds in the Erlong Lake did not pose a risk to human health. CDOM fluorescence might be a potential tool for monitoring PAE concentration and transport, as well as for rapid health risk assessments based on many experiments in the future.

## Figures and Tables

**Table 1:** Average concentrations of water quality parameters (*N* = 45) (AVESD).

Average	DOC	Chl-a	TP	NH_3_-N	COD	ISM	OSM
GB3838-2002	-	-	0.05	0.15	30	-	-
JUN 2017	26.38 ± 2.09	39.01 ± 13.33	0.02 ± 0.003	0.09 ± 0.06	28.28 ± 18.87	13.60 ± 8.37	4.05 ± 3.378
OCT 2017	23.63 ± 1.62	14.51 ± 7.52	0.03 ± 0.005	0.12 ± 0.07	22.57 ± 23.53	11.11 ± 3.32	1.20 ± 0.872

**Table 2:** Concentrations of phthalate esters (PAEs) in Erlong Lake (mg L^−1^).

PAEs	*N*	JUN				OCT				∑PAEs	%
		%	Avg.	Med.	Max.	%	Avg.	Med.	Max.	Avg. ± SD.	
DMP	45	4.0	0.013	0.013	0.032	1.1	0.006	0.006	0.009	0.01 ± 0.007	2.4
DEP	45	94.0	0.299	0.294	0.410	97.6	0.533	0.535	0.617	0.403 ± 0.130	95.9
DBP	45	1.9	0.006	0.006	0.016	1.3	0.007	0.006	0.033	0.007 ± 0.005	1.7
∑3PAEs	135	100	0.318			100	0.546			0.420.158	100

**Table 3:** Chromophoric-dissolved organic matter (CDOM) absorption parameters from water samples collected in the Erlong Lake (Avg. ± SD).

Time	*N*	*FI*_370_	*FI*_310_	*S*_275–295_	*a*(350)	*a*(254)	*E*_250:365_	*SUVA*_254_
JUN	25	1.39 ± 0.07	1.04 ± 0.08	0.26 ± 0.10	2.21 ± 1.18	16.06 ± 8.15	19.36 ± 4.85	0.62 ± 0.31
OCT	20	1.14 ± 0.03	0.81 ± 0.03	0.24 ± 0.12	4.73 ± 1.60	23.04 ± 7.72	6.32 ± 0.21	1.08 ± 0.39

Units of the absorption coefficients of CDOM are m^−1^, *S*_275–295_ is nm^−1^, and *SUVA*_254_ is L·mg·C^−1^·m^−1^.

**Table 4:** Pearson correlation analysis between fluorescence intensities of integrated regions (*F*_R1_, *F*_R2_, *F*_R3_, *F*_R4_, and *F*_R5_).

Component	*F*_R1_	*F*_R2_	*F*_R3_	*F*_R4_	*F*_R5_
*F*_R1_	1.0 *	0.68 *	0.49 *	0.51 *	0.46 *
*F*_R2_		1.0 *	0.67 *	0.61 *	0.69 *
*F*_R3_			1.0 *	0.96 *	0.88 *
*F*_R4_				1.0 *	0.89 *
*F*_R5_					1.0 *

* *p* < 0.01 level.

**Table 5:** Pearson correlation analysis between water quality parameters and fluorescence regional integration (FRI) fluorescent components.

Component	*N*	*F*_R1_	*F*_R2_	*F*_R3_	*F*_R4_	*F*_R5_	*FI*_370_	*FI*_310_
Chl-a	45	0.29 *	0.48 *	0.57 *	0.50 *	0.44 *	0.40 *	0.49 *
DOC	45	0.45 *	0.48 *	0.68 *	0.68 *	0.58 *	0.63 *	0.59 *
*a*(350)	45	0.33 *	0.48 *	0.58	0.52 *	0.55 *	0.44 *	0.48 *

* *p* < 0.01 level.

**Table 6:** Correlation coefficients (*R^2^*) and significance levels (*p*) of two-tailed correlation between PAEs and FRI fluorescence components in Erlong Lake.

Component	N	R1	R2	R3	R4	R5	*FI*_370_
DEP	45	0.36 *	0.44 *	0.78 *	0.77 *	0.58 *	0.68 *
DMP	45	-	-	-	-	-	-
DBP	45	-	-	-	-	-	-
∑3PAEs	45	0.34 *	0.43 *	0.76 *	0.75 *	0.57 *	-

* *p* < 0.001 level.

## References

[1] Tranvik L.J., Downing J.A., Cotner J.B., Loiselle S.A., Striegl R.G., Ballatore T.J., Dillon P., Finlay K., Fortino K., Knoll L.B. (2009). Lakes and reservoirs as regulators of carbon cycling and climate. Limnol. Oceanogr..

[2] Zhao Y., Song K.S., Wen Z.D., Fang C., Shang Y.X., Lv L.L. (2017). Evaluation of CDOM sources and their links with water quality in the lakes of northeast china using fluorescence spectroscopy. J. Hydrol..

[3] Wang Z., Liu W., Zhao N., Li H., Zhang Y., Ma W.S., Liu J. (2007). Composition analysis of colored dissolved organic matter in Taihu Lake based on three dimension excitation–emission fluorescence matrix and PARAFAC model, and the potential application in water quality monitoring. J. Environ. Sci..

[4] Guo W.D., Yang L.Y., Zhai W.D., Chen W.Z., Osburn C.L., Huang X., Li Y. (2014). Runoff-mediated seasonal oscillation in the dynamics of dissolved organic matter in different branches of a large bifurcated estuary-The Changjiang Estuary. J. Geophys. Res. Biogeosci..

[5] Zhou Y., Jeppesen E., Zhang Y., Shi K., Liu X., Zhu G. (2016). Dissolved organic matter fluorescence at wavelength 275/342 nm as a key indicator for detection of point-source contamination in a large Chinese drinking water lake. Chemosphere.

[6] Li S.J., Zhang J.Q., Guo E.L., Zhang F., Ma Q.Y., Mu G.Y. (2017). Dynamics and ecological risk assessment of chromophoric dissolved organic matter in the Yinma River watershed: Rivers, reservoirs, and urban waters. Environ. Res..

[7] Zhao Y., Song K.S., Wen Z.D., Li L., Zang S.Y., Shao T.T., Li S.J., Du J. (2016). Seasonal characterization of CDOM for lakes in semiarid regions of Northeast China using excitation-emission matrix fluorescence and parallel factor analysis (EEM-PARAFAC). Biogeosci. Discuss..

[8] De Laurentiis E., Minella M., Maurino V., Minero C., Brigante M., Mailhot G., Vione D. (2012). Photochemical production of organic matter triplet states in water samples from mountain lakes, located below or above the tree line. Chemosphere.

[9] Zhang Y.L., Yin Y., Feng L.Q., Zhu G.W., Shi Z.Q., Liu X.H., Zhang Y.Z. (2011). Characterizing chromophoric dissolved organic matter in Lake Tianmuhu and its catchment basin using excitation-emission matrix fluorescence and parallel factor analysis. Water Res..

[10] Zhao Y., Song K.S., Li S.J., Ma J.H., Wen Z.D. (2016). Characterization of CDOM from urban waters in Northern-Northeastern China using excitation-emission matrix fluorescence and parallel factor analysis. Environ. Sci. Pollut. Res..

[11] Zhao Y., Song K., Shang Y., Shao T., Wen Z., Lv L. (2017). Characterization of CDOM of river waters in China using fluorescence excitation-emission matrix and regional integration techniques. J. Geophys. Res. Biogeosci..

[12] Chen W., Westerhoff P., Leenheer J.A., Booksh K. (2003). Fluorescence excitation-emission matrix regional integration to quantify spectra for dissolved organic matter. Environ. Sci. Technol..

[13] Bui T.T., Giovanoulis G., Cousins A.P., Magnér J., Cousins I.T., de Wit C.A. (2016). Human exposure, hazard and risk of alternative plasticizers to phthalate esters. Sci. Total Environ..

[14] Wang H., Liang H., Gao D.W. (2017). Occurrence and risk assessment of phthalate esters (PAEs) in agricultural soils of the Sanjiang plain, Northeast China. Environ. Sci. Pollut. Res..

[15] Li C., Chen J.Y., Wang J.H., Han P., Luan Y.X., Ma X.P., Lu A.X. (2016). Phthalate esters in soil, plastic film, and vegetable from greenhouse vegetable production bases in Beijing, China: Concentrations, sources, and risk assessment. Sci. Total Environ..

[16] Kong Y., Shen J., Chen Z., Kang J., Li T., Wu X., Fan L. (2017). Profiles and risk assessment of phthalate acid esters (PAEs) in drinking water sources and treatment plants, East China. Environ. Sci. Pollut. Res..

[17] Williamson C.E., Brentrup J.A., Zhang J., Renwick W.H., Hargreaves B.R., Knoll L.B., Overholt E.P., Rose K.C. (2014). Lakes as sensors in the landscape: Optical metrics as scalable sentinel responses to climate change. Limnol. Oceanogr..

[18] Wang N., Zhang H.Y., Wang H.L., Zhang Z.X. (2004). Spatial analysis of soil erosion and non-point source pollution based on GIS in Erlong lake watershed, Jilin province. Chin. Geogr. Sci..

[19] Song K.S., Li L., Wang Z.D., Liu D.W., Zhang B., Xu J.P., Du J., Li L.H., Li S., Wang Y.D. (2012). Retrieval of total suspended matter (TSM) and chlorophyll-a (Chl-a) concentration from remote-sensing data for drinking water resources. Environ. Monit. Assess..

[20] Li S.J., Chen Y.N., Zhang J.Q., Song K.S., Mu G.Y., Sun C.Y., Ju H.Y., Ji M.C. (2017). The relationship of chromophoric dissolved organic matter parallel factor analysis fluorescence and polycyclic aromatic hydrocarbons in natural surface waters. Environ. Sci. Pollut. Res..

[21] Stedmon C.A., Amon R.M.W., Rinehart A.J., Walker S.A. (2011). The supply and char-acteristics of colored dissolved organic matter (CDOM) in the Arctic Ocean: Pan Arctic trends and differences. Mar. Chem..

[22] Mcknight D.M., Boyer E.W., Westerhoff P.K., Doran P.T., Kulbe T., Andersen D.T. (2001). Spectrofluorometric characterization of dissolved organic matter for indication of precursor organic material and aromaticity. Limnol. Oceanogr..

[23] Zhang Y.L., Zhang E.L., Yin Y., VanDijk M.A., Feng L.Q., Shi Z.Q., Liu M.L., Qin B.Q. (2010). Characteristics and sources of chromophoric dissolved organic matter in lakes of the Yungui Plateau, China, differing in trophic state and altitude. Limnol. Oceanogr..

[24] Chen X., Zhou W.Q., Steward T.A.P., Li W.F., Han L.J. (2016). Spatial-Temporal Variations of Water Quality and Its Relationship to Land Use and Land Cover in Beijing, China. Int. J. Environ. Res. Public Health.

[25] Li S.J., Zhang J.Q., Mu G.Y., Ju H.Y., Wang R., Li D.J., Shabbir A.H. (2016). Spatiotemporal Characterization of Chromophoric Dissolved Organic Matter (CDOM) and CDOM-DOC Relationships for Highly Polluted Rivers. Water.

[26] Worrall F., Burt T.P. (2008). The effects of severe drought on the dissolved organic carbon (DOC) concentration and flux from British rivers. J. Hydrol..

[27] He W., Qin N., Kong X., Liu W., He Q., Ouyang H., Yang C., Jiang Y., Wang Q., Yang B. (2013). Spatio-temporal distributions and the ecological and health risks of phthalate esters (PAEs) in the surface water of a large, shallow Chinese lake. Sci. Total Environ..

[28] He X.S., Xi B.D., Li X., Pan H.W., An D., Bai S.G., Li D., Cui D.Y. (2013). Fluorescence excitation-emission matrix spectra coupled with parallel factor and regional integration analysis to characterize organic matter humification. Chemosphere.

[29] Yu H.B., Song Y.H., Gao H.J., Liu L., Yao L.L., Peng J.F. (2015). Applying fluorescence spectroscopy and multivariable analysis to characterize structural composition of dissolved organic matter and its correlation with water quality in an urban river. Environ. Earth Sci..

[30] Jaffé R., Boyer J.N., Lu X., Maie N., Yang C., Scully N.M., Mock S. (2004). Source characterization of dissolved organic matter in subtropical mangrove-dominated estuary by fluorescence analysis. Mar. Chem..

[31] Gonnelli M., Vestri S., Santinelli C. (2013). Chromophoric dissolved organic matter and microbial enzymatic activity. A biophysical approach to understand the marine carbon cycle. Biophys. Chem..

[32] Weishaar J.L., Aiken G.R., Bergamaschi B.A., Fram M.S., Fujii R., Mopper K. (2003). Evaluation of specific ultraviolet absorbance as an indicator of the chemical composition and reactivity of dissolved organic carbon. Environ. Sci. Technol..

[33] Cory R.M., McKnight D.M., Chin Y.P., Miller P., Jaros C.L. (2007). Chemical characteristics of fulvic acids from Arctic surface waters: Microbial contributions and photochemical transformations. J. Geophys. Res. Biogeosci..

[34] Cui H., Shi J.H., Qiu L.L., Zhao Y., Wei Z.M., Wang X.L., Jia L.M., Li J.M. (2016). Characterization of chromophoric dissolved organic matter and relationships among PARAFAC components and water quality parameters in Heilongjiang, China. Environ. Sci. Pollut. Res..

[35] Wang J., Chen G., Christie P., Zhang M., Luo Y., Teng Y. (2015). Occurrence and risk assessment of phthalate esters (PAEs) in vegetables and soils of suburban plastic film greenhouses. Sci. Total Environ..

